# Predictive Factors of Antibody–Drug Conjugate Treatment in Metastatic Breast Cancer: A Narrative Review

**DOI:** 10.3390/cancers16234082

**Published:** 2024-12-05

**Authors:** Gennaro Gadaleta-Caldarola, Laura Lanotte, Anna Natalizia Santoro, Antonello Pinto, Arianna Gadaleta-Caldarola, Luca Giacomelli, Palma Fedele

**Affiliations:** 1 Oncology Unit, “Mons. A. R. Dimiccoli” Hospital, 70051 Barletta, Italy; gennaro.gadaleta@aslbat.it (G.G.-C.); laura.lanotte@aslbat.it (L.L.); 2 Oncology Unit, “Dario Camberlingo” Hospital, 72021 Francavilla Fontana, Italy; annanatalizia.santoro@asl.brindisi.it (A.N.S.); antonello.pinto@studenti.unimi.it (A.P.); palma.fedele@asl.brindisi.it (P.F.); 3 Medicine and Surgery, Università degli Studi di Siena, 53100 Siena, Italy; arianna.gadaletac@gmail.com; 4 Polistudium SRL, 20121 Milan, Italy

**Keywords:** antibody–drug conjugates, metastatic breast cancer, predictive factors, HER2, TROP-2, trastuzumab emtansine, trastuzumab deruxtecan, sacituzumab govitecan

## Abstract

Antibody–drug conjugates (ADCs) have revolutionized the treatment landscape for metastatic breast cancer, offering targeted delivery of cytotoxic agents with improved efficacy and tolerability compared to conventional chemotherapy. This narrative review explores key predictive factors influencing the efficacy of ADCs, focusing on HER2-targeted therapies, such as trastuzumab emtansine and trastuzumab deruxtecan, as well as sacituzumab govitecan for triple-negative breast cancer. HER2 expression, TROP-2 levels, hormone receptor status, and the tumor microenvironment emerge as critical biomarkers for patient selection and therapeutic outcomes. Additionally, we discuss resistance mechanisms, such as antigen loss, impaired drug internalization, and the role of circulating tumor DNA in predicting ADC response. Finally, future perspectives on the sequential use of ADCs and potential combination therapies are highlighted, along with emerging agents targeting alternative antigens like HER3 and LIV-1. Overall, identifying predictive biomarkers and overcoming resistance mechanisms are essential for optimizing the use of ADCs in metastatic breast cancer, thereby improving patient outcomes.

## Simple Summary

Antibody–drug conjugates (ADCs) are a cutting-edge treatment for metastatic breast cancer, combining targeted therapy with chemotherapy to attack cancer cells more precisely. However, their success varies, and understanding what makes them effective for some patients but less so for others is critical. This review identifies key factors, like specific proteins on cancer cells, genetic changes, and the tumor environment, that influence ADC effectiveness. It also explores challenges like resistance to treatment and ways to overcome them, such as developing new ADCs and combining them with other therapies. These insights aim to refine patient care by personalizing ADC treatments, ultimately improving outcomes for individuals with advanced breast cancer.

## 1. Introduction

Antibody–drug conjugates (ADCs) represent a major advance in breast cancer (BC) treatment, combining the specificity of monoclonal antibodies with the cytotoxic power of chemotherapy. By delivering cytotoxic payloads directly to cancer cells, ADCs minimize damage to normal tissues, offering a promising alternative to conventional treatments [1,2].

ADCs are composed of three key elements: a monoclonal antibody targeting cancer cell antigens, a linker, and a potent cytotoxic payload [3,4]. Some, such as trastuzumab deruxetan (T-DXd), exhibit a “bystander effect”, where the payload affects neighboring cells lacking the target antigen [1,2]. This selective delivery enhances tolerability and effectiveness compared to traditional chemotherapy [5].

The first FDA-approved ADC for metastatic BC (MBC), trastuzumab emtansine (T-DM1), remains essential for HER2-positive MBC, particularly in patients progressing after trastuzumab-based therapies [6,7,8,9]. T-DXd has shown superior efficacy over T-DM1, especially in human epidermal growth factor receptor 2 (HER2)-low and HER2-positive diseases [10,11,12]. Another notable ADC, sacituzumab govitecan (SG), targets TROP-2 and has demonstrated substantial benefit in triple-negative BC (TNBC), offering hope for heavily pretreated patients [13].

However, the success of ADCs varies among patients. Predictive factors, such as antigen expression, tumor microenvironment, and genetic mutations, are crucial for optimizing ADC use. Identifying reliable biomarkers and resistance mechanisms is essential for tailoring treatments to individual patients [2,14]. Emerging biomarkers, such as immune signatures, tumor mutation burden, and circulating tumor DNA (ctDNA), show potential for predicting ADC response and monitoring treatment resistance in real time [1,14,15]. These advancements hold promise for refining patient selection and improving outcomes in MBC.

This narrative review aims to synthesize current knowledge on predictive factors influencing the efficacy of ADCs, including key biomarkers, such as HER2 and TROP-2 expression, hormone receptor (HR) status, and the tumor microenvironment. Additionally, we summarize resistance mechanisms that limit ADC effectiveness and explore future perspectives, including the development of novel agents and therapeutic strategies to optimize ADC use in MBC treatment.

## 2. Key Predictive Biomarkers for ADC Efficacy

### 2.1. HER2 Expression and Amplification

HER2 is a critical biomarker in BC, found to be overexpressed in 15–20% of cases of MBC [16]. This amplification leads to uncontrolled cell proliferation through the activation of downstream signaling pathways, particularly PI3K–AKT and MAPK, contributing to an aggressive clinical course. HER2 status, determined via immunohistochemistry (IHC) and fluorescence in situ hybridization, serves as a key predictive factor for selecting patients who will benefit from HER2-targeted therapies (Table 1) [17,18].

T-DM1 was approved by the FDA as a second-line treatment for HER2-positive MBC following the phase III EMILIA trial, which showed a significant improvement in median overall survival (mOS; 30.9 vs. 25.1 months, hazard ratio [HR] 0.65) and an objective response rate (ORR) of 43.6% (vs. 30.8%) [19]. T-DXd was FDA-approved in 2020 for advanced HER2-positive MBC after at least two prior therapies. The phase II DESTINY-Breast01 trial showed an ORR of 61.4% and a median progression-free survival (mPFS) of 19.4 months [10]. In the phase III DESTINY-Breast03 trial, T-DXd was superior to T-DM1, with a mPFS of 28.8 vs. 6.8 months (HR 0.33) and a higher ORR (79.7% vs. 34.2%) [20]. T-DXd also showed efficacy in HER2-low patients, with an ORR of 37% and mPFS of 11.1 months [20]. A recently updated analysis confirmed the efficacy of T-DXd over T-DM1 on mPFS (28.8 vs. 6.8 months, HR 0.33), as well as on mOS (not reached in either group, HR 0.64) [21]. The phase III DESTINY-Breast04 trial showed T-DXd’s superiority over chemotherapy in HER2-low MBC, with mPFS of 9.9 vs. 5.1 months (HR 0.50) and mOS of 23.4 vs. 16.8 months (HR 0.64). T-DXd also improved outcomes in HR-positive BC and TNBC subgroups [11]. FDA approval for T-DXd in HER2-low MBC is indicated for patients who have previously undergone chemotherapy in the metastatic setting or experienced disease recurrence during or within 6 months of completing adjuvant chemotherapy.

Overall, HER2 status remains a critical predictive biomarker for the response to T-DM1 and T-DXd. While T-DM1 is effective in HER2-positive tumors, T-DXd’s broader applicability to HER2-low cancers provides a significant advancement, particularly for patients with reduced HER2 expression or those who have developed resistance to earlier HER2-targeted therapies [15,18].

### 2.2. Trop-2 Expression in Triple-Negative Breast Cancer

SG is an ADC comprising an anti-TROP2 mAb linked to the cytotoxic agent SN-38 via a cleavable linker (DAR, 7.6–8:1) [22]. In April 2021, the FDA approved SG (10 mg/kg) for metastatic TNBC after ≥2 prior systemic therapies, based on the phase III ASCENT trial [23]. This trial compared SG to treatment of physician’s choice (TPC) in 468 patients, showing an ORR of 35% (vs. 5%), a longer mPFS (5.6 vs. 1.7 months; HR 0.41), and mOS (12.1 vs. 6.7 months; HR 0.48). Although TROP-2 expression was not required for enrollment, higher efficacy was seen in TROP-2-high and TROP-2-median tumors [23]. Indeed, an exploratory biomarker analysis from the ASCENT trial showed that patients with high and medium TROP-2 expression treated with SG had improved outcomes compared to those with low TROP-2 expression [23]. Specifically, patients with high TROP-2 expression had a mPFS of 6.9 months compared to 2.5 months in the TPC arm, while those with low TROP-2 expression had an mPFS of 2.7 months (vs. 1.6 months in the TPC arm) [23]. mOS was also higher in patients with high and medium TROP-2 expression (14.2 and 14.9 months, respectively) compared to 9.3 months in patients with low TROP-2 expression [23]. Thus, while SG benefits all patients, it appears particularly effective in tumors with higher TROP-2 expression levels.

## 3. Other Predictive Factors

### 3.1. Predictive Factors for T-DM1 Efficacy

While T-DM1 has demonstrated efficacy in HER2-positive MBC, response to treatment varies significantly across patients due to several predictive factors. Identifying these factors is crucial for optimizing patient selection and treatment outcomes.

#### 3.1.1. HER2 Expression and Amplification

HER2 amplification remains the primary biomarker guiding the use of T-DM1. However, variations in HER2 levels can influence treatment outcomes. Patients with low HER2 expression or HER2 mutations are less likely to respond to T-DM1, leading to primary resistance [24].

#### 3.1.2. ctDNA and Genetic Mutations

The presence of HER2 amplification in ctDNA is emerging as a predictive biomarker for T-DM1 efficacy. In a study by Sakai et al. [24], patients with detectable HER2 amplification in ctDNA prior to T-DM1 treatment had significantly better responses than those without such amplification [24]. Moreover, patients harboring *PIK3CA* mutations in ctDNA were more likely to exhibit primary resistance to T-DM1, suggesting that genomic alterations in tumor DNA can serve as indicators of reduced efficacy.

#### 3.1.3. HR Status

The impact of HR status on T-DM1 efficacy is another important consideration. Patients with HR-positive, HER2-positive BC often show a less favorable response to T-DM1 compared to HR-negative patients. Moinard et al. [25] found that HR-positive status was associated with shorter PFS and OS in patients receiving T-DM1 as second-line therapy [26]. Furthermore, ER positivity, in combination with lower HER2 levels, has been linked to primary resistance, as shown by Watanuki et al., further emphasizing the need for careful patient stratification based on HR status [26].

#### 3.1.4. Pharmacokinetic Factors and Exposure–Response Relationships

Pharmacokinetic studies have indicated that patients with higher T-DM1 plasma exposure, particularly those with higher minimum concentrations after the first cycle, tend to have better outcomes in terms of PFS and OS [27]. This relationship between T-DM1 exposure and efficacy suggests that optimizing dosage based on pharmacokinetic profiles could further personalize therapy. In contrast, Li et al. reported that patients in the lowest quartile of T-DM1 exposure had similar or better outcomes compared to controls, underscoring the complex nature of exposure–response relationships [28].

#### 3.1.5. Previous Treatment and Resistance Mechanisms

Prior exposure to other HER2-targeted therapies, such as trastuzumab and pertuzumab, also affects T-DM1 efficacy. For instance, patients who progress on dual HER2 blockade often exhibit lower response rates to T-DM1, potentially due to cross-resistance mechanisms [25]. In this context, Sakai et al. highlighted the role of alternative signaling pathways, such as PI3K/AKT, which may drive resistance and diminish T-DM1 effectiveness [24].

### 3.2. Predictive Factors for T-DXd Efficacy

Several factors influence the efficacy of T-DXd, and understanding these predictors is critical for optimizing patient outcomes.

#### 3.2.1. HER2 Expression and Amplification

In the phase II DAISY trial, T-DXd demonstrated a confirmed ORR of 70.6% in patients with HER2-overexpressing mBC (HER2 IHC 3+ or ERBB2 in situ hybridization-positive) [29]. This efficacy extended to patients with lower HER2 expression, with an ORR of 37.5% in HER2-low mBC (IHC 1+ or IHC 2+/ISH-negative). Interestingly, even in HER2-non-expressing tumors (HER2 IHC 0), T-DXd elicited a confirmed ORR of 29.7%, demonstrating some activity in the absence of HER2 expression, although this rate was lower compared to HER2-expressing cohorts [29].

This heterogeneity in response highlights that while HER2 expression is a strong predictor of T-DXd efficacy, the drug’s activity may also rely on mechanisms beyond HER2 targeting, such as the “bystander effect”, where the cytotoxic payload can kill neighboring cells not expressing HER2 [30]. Nonetheless, higher HER2 expression correlates with better outcomes; in the DAISY trial, patients with HER2 IHC 3+ experienced superior PFS compared to those with HER2-low or HER2-negative tumors (median PFS: 11.1 months vs. 6.7 months and 4.2 months, respectively) [29].

These findings underscore the importance of HER2 testing in patient stratification for T-DXd therapy, especially as HER2-low patients represent a growing population that can benefit from this therapy, expanding its use beyond traditionally HER2-positive mBC [30].

#### 3.2.2. Brain Metastases

Brain metastases (BMs) are a common complication in MBC, affecting up to 50% of patients, and significantly influence prognosis and treatment outcomes [31,32]. T-DXd has demonstrated substantial intracranial activity in both clinical trials and real-world studies, providing a new therapeutic avenue for patients with HER2-positive mBC and BMs. In the DESTINY-Breast03 trial, T-DXd significantly improved PFS in patients with BMs compared to T-DM1, with a median PFS of 15.0 months versus 3.0 months (HR 0.25) [31]. Moreover, T-DXd achieved an intracranial objective response rate (ORR) of 65.7% in this population, compared to 34.3% for T-DM1 [31]. Further supporting these findings, the pooled analysis of the DESTINY-Breast01, 02, and 03 trials demonstrated a similar ORR of 45.5% in patients with untreated/active BMs and 45.2% in those with treated/stable BMs [33]. The median central nervous system PFS in these groups was 18.5 months for untreated BMs and 12.3 months for treated BMs, showing that T-DXd is highly effective across different BM treatment statuses [33]. The TUXEDO-1 trial, focusing on patients with HER2-positive mBC and active BMs, showed even more striking results, with a median PFS of 21 months and an intracranial ORR of 73.3%, further underscoring the drug’s efficacy in controlling brain lesions [34]. Additionally, real-world studies, such as the one by Dannehl et al., have shown that T-DXd achieved an intracranial disease control rate of 88% in patients with stable and active BMs, with a median intracranial PFS of 11.2 months [35].

Data from the DEBBRAH trial also provide insight into T-DXd’s efficacy in HER2-low mBC BM, a population with limited treatment options. In patients with active BMs, the intracranial ORR was 33.3%, and the median intracranial duration of response was 7.2 months, demonstrating that T-DXd may offer benefit even in HER2-low disease [36].

Collectively, these findings position T-DXd as a key systemic therapy for patients with HER2-positive mBC and BMs, offering durable intracranial control and survival benefits, even in patients with untreated or progressing brain lesions [32,37].

#### 3.2.3. HR Status

The HR status significantly influences the response to T-DXd. In the DE-REAL study, which included 143 patients, 75% had HR-positive/HER2-positive tumors, and 25% had HR-negative/HER2-positive disease. The efficacy of T-DXd was consistent regardless of HR status, with a median PFS of 17 months for HR-positive and 15 months for HR-negative patients, indicating no statistically significant difference (HR 0.92; 95% CI, 0.51–1.67; *p* = 0.78) [38].

Furthermore, the ORR was comparable between the two groups, with no substantial variation in disease control. This suggests that T-DXd’s effectiveness in treating HER2-positive mBC is robust across different HR statuses, offering a reliable therapeutic option for both HR-positive and HR-negative patients. These findings highlight T-DXd’s broad applicability in diverse patient populations, emphasizing its utility as a key treatment option irrespective of HR expression.

### 3.3. Predictive Factors for SG Efficacy

#### 3.3.1. Previous Therapy

In the phase I/II IMMU-132-01 trial, SG demonstrated an ORR of 31.5%, an mPFS of 5.5 months, and an mOS of 12.0 months in patients with HR+/HER2- mBC who had progressed on prior endocrine and chemotherapy treatments [39]. Additionally, it exhibited a favorable safety profile, with neutropenia being the most common grade 3 or higher adverse event [39].

#### 3.3.2. BMs

Real-world evidence and retrospective analyses have also highlighted the efficacy of SG in mBC patients with BMs. In a multicenter real-world study, SG achieved an intracranial disease control rate of 42% in patients with BM, with a median intracranial PFS of 2.7 months, demonstrating some intracranial efficacy even in heavily pretreated patients [35].

#### 3.3.3. Sequence of Treatment

The sequential use of SG and T-DXd in HER2-low mBC, though currently recommended, has yielded limited clinical benefit in most patients. A retrospective analysis (ADC-Low study) showed that median PFS was short, with 2.7 months for SG when used after T-DXd in HER2-low patients, indicating that while sequential administration is feasible, additional research is needed to identify patients who may benefit from these ADCs when used sequentially [40].

Table 2 summarizes the above.

## 4. Future Perspectives and Lines of Research

The future of ADCs in MBC treatment focuses on overcoming challenges related to the tumor microenvironment (TME), and resistance mechanisms, and optimizing therapeutic sequencing and combination strategies (Table 3) [2,14,22,41,42,43]. Emerging ADCs also hold significant promise for expanding treatment options.

### 4.1. Role of the TME

The TME plays a crucial role in the efficacy of ADCs, influencing both drug delivery and immune response [41,43]. Tumor heterogeneity, varying antigen expression, and the presence of stromal components can limit ADC penetration. Components, such as stromal fibroblasts, tumor-associated macrophages, and extracellular matrix, can act as physical barriers that hinder ADC efficacy.

### 4.2. Resistance to ADCs

Resistance to ADCs remains a significant hurdle [14]. Mechanisms include antigen loss or downregulation, alterations in drug internalization, and efflux of cytotoxic payloads. Resistance to T-DM1 and T-DXd, for instance, may be linked to lower HER2 expression or mutations in HER2 itself, as well as upregulation of drug efflux transporters, such as ABCG2. Efforts to mitigate resistance include designing ADCs with improved linkers and payloads that bypass common resistance mechanisms, such as payloads that are less dependent on target internalization.

### 4.3. Sequential Treatment of ADCs

Sequencing ADCs with different mechanisms of action is a critical research area [2,42]. Changing the payload (e.g., switching from T-DM1 to T-DXd) may have outcomes, with some evidence showing better PFS when using distinct ADCs sequentially. However, cross-resistance remains a concern, particularly when ADCs share similar targets or mechanisms of action. The optimal sequence and combination of ADCs, especially in HER2-low and HER2-positive populations, remains a major goal for future research.

### 4.4. Combination Strategies and Emerging ADCs

Combining ADCs with other therapeutic agents, such as immune checkpoint inhibitors, PARP inhibitors, and cyclin-dependent kinase 4/6 (CDK4/6) inhibitors, offers an avenue to improve response rates and overcome resistance [22]. Additionally, co-targeting TME components along with ADCs is an emerging strategy to boost anti-tumor efficacy. Radiolabeled ADCs represent another promising direction, enabling both molecular imaging and therapeutic effects [44,45,46]. These approaches, including the use of radionuclides such as Lutetium-177 and Zirconium-89, enhance the precision of ADCs while offering theranostic potential in HER2-positive BC.

Furthermore, new ADCs targeting antigens beyond HER2 and TROP-2, such as HER3, LIV-1, B7-H4, and nectin-4, are in various stages of development. These next-generation ADCs utilize innovative linkers and cytotoxic agents, expanding therapeutic possibilities to additional subtypes of BC, including TNBC and HR-positive, HER2-negative subtypes.

## 5. Concluding Remarks

**Figure 1:**
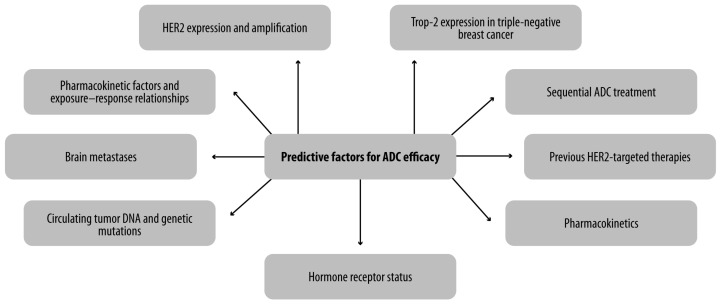
Predictive factors for ADCs efficacy. ADC: Antibody–drug conjugate.

ADCs represent a significant advancement in the treatment of MBC, particularly in subtypes like HER2-positive, HER2-low, and TNBC. Predictive biomarkers, such as HER2 and TROP-2 expression, HR status, and the tumor microenvironment, are crucial in guiding therapy and optimizing treatment outcomes (Figure 1). Novel ADCs, such as T-DXd and SG, have shown promising efficacy, even in heavily pretreated patients, expanding the therapeutic arsenal. However, the use of ADCs is now without its limitations. Antigen heterogeneity can reduce efficacy in tumors with low or variable antigen expression. Off-target effects and systemic toxicities remain concerns due to the unintended release of the cytotoxic payload. Resistance mechanisms, including antigen loss and efflux of the payload, further limit their effectiveness. Additionally, the high cost of ADCs challenges accessibility, particularly in resource-limited settings. Overcoming these hurdles through next-generation ADCs, improved patient selection, and novel combination strategies is crucial to maximize their potential.

Indeed, Future clinical directions include the development of next-generation ADCs targeting alternative antigens, such as HER3 and LIV-1, to address unmet needs in BC subtypes with limited treatment options. Combining ADCs with synergistic agents, such as immune checkpoint inhibitors or PARP inhibitors, and the use of radiolabeled ADCs represent a promising strategy to enhance efficacy and overcome resistance. Furthermore, leveraging ADCs in earlier stages of BC, such as the neoadjuvant or adjuvant settings, could improve long-term outcomes and prevent disease progression. Continued research into resistance mechanisms and personalized treatment approaches will be critical for broadening the applicability of ADCs and achieving durable responses in diverse patient populations.

## Figures and Tables

**Table 1:** Summary of key findings from pivotal clinical trials evaluating T-DM1 and T-DXd in HER2-positive and HER2-low MBC.

Study	Trial Name	Therapy	Patient Population	Key Findings	References
Verma et al. (2012)	EMILIA (phase III)	T-DM1	HER2-positive MBC, previously treated	mOS: 30.9 vs. 25.1 months (HR 0.65) ORR: 43.6% vs. 30.8%	[19]
Modi et al. (2020)	DESTINY-Breast01 (phase II)	T-DXd	Advanced HER2-positive MBC, ≥2 prior therapies	ORR: 61.4% mPFS: 19.4 months	[10]
Cortés et al. (2022)	DESTINY-Breast03 (phase III)	T-DXd vs. T-DM1	HER2-positive MBC, previously treated	mPFS: 28.8 vs. 6.8 months (HR 0.33) ORR: 79.7% vs. 34.2% HER2-low ORR: 37%, mPFS: 11.1 months	[20]
Hurvitz et al. (2023)	DESTINY-Breast03 (update)	T-DXd vs. T-DM1	HER2-positive MBC	mPFS: 28.8 vs. 6.8 months (HR 0.33) mOS: Not reached in either group (HR 0.64)	[21]
Modi et al. (2022)	DESTINY-Breast04 (phase III)	T-DXd	HER2-low MBC	mPFS: 9.9 vs. 5.1 months (HR 0.50) mOS: 23.4 vs. 16.8 months (HR 0.64)	[11]

HR: Hazard ratio; mOS: Median overall survival; mPFS: Median progression-free survival; MBC: Metastatic breast cancer; ORR: Objective response rate; T-DM1: Trastuzumab emtansine; T-DXd: Trastuzumab deruxtecan.

**Table 2:** Other predictive factors influencing the efficacy of antibody–drug conjugates (T-DM1, T-DXd, and sacituzumab govitecan) in the treatment of MBC.

Predictive Factor	Key Details	Effect	References
HER2 expression and amplification	High HER2 expression (IHC 3+) is linked to better outcomes. Low HER2 or mutations may lead to resistance.	Strong predictor for T-DM1 efficacy. T-DXd shows activity in HER2-low and HER2-negative tumors.	[24,29]
ctDNA	HER2 amplification in ctDNA predicts better responses. PIK3CA mutations in ctDNA signal resistance.	ctDNA analysis helps predict T-DM1 efficacy and resistance.	[24]
HR status	HR-positive patients show shorter PFS/OS compared to HR-negative. ER-positive, low HER2 expression leads to resistance.	T-DM1 efficacy is reduced in HR-positive patients. T-DXd efficacy is unaffected by HR status.	[25,38]
Pharmacokinetics	Higher T-DM1 plasma exposure is linked to better outcomes. Complex relationship between dose and response.	Personalizing dosage based on pharmacokinetic profiles may optimize outcomes.	[27,28]
Previous HER2-targeted therapies	Prior exposure to trastuzumab and pertuzumab reduces T-DM1 efficacy due to cross-resistance.	Alternative signaling pathways may reduce efficacy after dual HER2 blockade.	[24,25]
Brain metastases	T-DXd significantly improves PFS and intracranial activity in HER2-positive and HER2-low patients with brain metastases.	A key factor influencing T-DXd efficacy. Substantial intracranial control with T-DXd.	[31,34]
TROP-2 expression	Higher TROP-2 expression correlates with better responses, but low TROP-2 expression also benefits.	Predictor of sacituzumab govitecan efficacy, especially in TNBC.	[23]
Sequential ADC treatment	Limited clinical benefit for sacituzumab govitecan after T-DXd in HER2-low patients.More research needed	for sequential use of ADCs.	[40]

ADC: Antibody–drug conjugate; ctDNA: Circulating tumor DNA; HER2: Human epidermal growth factor receptor 2; HR: Hormone receptor; IHC: Immunohistochemistry; MBC: Metastatic breast cancer; PFS: Progression-free survival; ORR: Objective response rate; T-DM1: Trastuzumab emtansine; T-DXd: Trastuzumab deruxtecan; TNBC: Triple-negative breast cancer.

**Table 3:** Open lines of research on ADCs in MBC.

Key Concept	Details
Tumor microenvironment	The TME influences ADC efficacy by affecting drug delivery and immune response. Stromal components, such as fibroblasts, macrophages, and extracellular matrix, act as barriers to ADC penetration.
Resistance mechanisms	Resistance arises from antigen loss/downregulation, impaired drug internalization, and upregulation of efflux transporters (e.g., ABCG2). Resistance to T-DM1 and T-DXd can be caused by decreased HER2 expression or mutations.
Sequential ADC treatment	Sequencing ADCs with different mechanisms (e.g., switching from T-DM1 to T-DXd) may improve outcomes. However, cross-resistance is a concern, particularly when ADCs share similar targets or mechanisms.
Combination strategies	Combining ADCs with agents, such as immune checkpoint inhibitors, PARP inhibitors, and CDK4/6 inhibitors, may enhance efficacy and overcome resistance. Co-targeting the TME is another promising strategy.
Emerging ADCs	New ADCs targeting antigens, such as HER3, LIV-1, B7-H4, and nectin-4, are under development. These ADCs have innovative linkers and cytotoxic agents, offering therapeutic potential for TNBC and HER2-negative breast cancers.
Radiolabeled ADCs	Combine radionuclides (e.g., lutetium-177, zirconium-89) with ADCs to enable imaging and therapeutic use.

ADC: Antibody–drug conjugate; HER2: Human epidermal growth factor receptor 2; MBC: Metastatic breast cancer; PARP: Poly (ADP-ribose) polymerase; T-DM1: Trastuzumab emtansine; T-DXd: Trastuzumab deruxtecan; TME: Tumor microenvironment; TNBC: Triple-negative breast cancer.
